# Impact of Clonal Variability on Phenolics and Radical Scavenging Activity of Grapes and Wines: A Study on the Recently Developed Merlot and Cabernet Franc Clones (*Vitis vinifera* L.)

**DOI:** 10.1371/journal.pone.0163823

**Published:** 2016-10-12

**Authors:** Milica Pantelić, Dragana Dabić Zagorac, Maja Natić, Uroš Gašić, Sonja Jović, Dragan Vujović, Jelena Popović Djordjević

**Affiliations:** 1 Innovation Center, Faculty of Chemistry Ltd, University of Belgrade, Belgrade, Serbia; 2 Faculty of Chemistry, University of Belgrade, Belgrade, Serbia; 3 ODPF "Radmilovac", Faculty of Agriculture, University of Belgrade, Belgrade, Serbia; 4 Department of Horticulture, Faculty of Agriculture, University of Belgrade, Belgrade, Serbia; 5 Department of Food Technology and Biochemistry, Faculty of Agriculture, University of Belgrade, Belgrade, Serbia; National Institute for Agronomic Research, FRANCE

## Abstract

In this study, grapes and corresponding wines of Merlot /No 022, 025 and 029/ and Cabernet Franc /No 02, 010 and 012/ clones (recently developed) were evaluated regarding the total phenolic content, total anthocyanin content, and radical scavenging activity, aiming to better understand their quality and market potential. The nineteen individual polyphenols were quantified in studied grape samples using UHPLC coupled to a triple-quadrupole mass spectrometer. The mother grapes and wines were used as the relevant standards. In the grape, studied characteristics were monitored at three stages of berry development: green berry, véraison and mature berry. The mature grape of clones presented high values of total phenolics (3.81–10.89 g gallic acid equivalent kg^-1^ frozen weight), anthocyanin content (359.00–1668.18 mg malvidin-3-*O*-glucoside kg^-1^ frozen weight) and the radical scavenging activity (41.37–80.48 mmol trolox equivalent kg^-1^ frozen weight) depending on the clone. Grapes and wines of Merlot No 025 and Cabernet Franc No 010 stood out with the highest values of all three parameters. Generally, the high correlation was observed between TPC and RSA values for green berries and mature grapes, as well as for wine samples. The most abundant phenolics in both Merlot and Cabernet Franc grapes were gallic acid, *p*-hydroxybenzoic acid, ferulic acid, catechin, epicatechin, gallocatechin gallate, catechin gallate, and rutin. Catechin, epicatechin, epigallocatechin, and catechin gallate, typical for the ripe grape of Merlot and Cabernet Franc clones, showed significant correlation with RSA values. Concentrations of individual polyphenols varied depending on the sample being studied and berry development stages. Merlot No 025 and Cabernet Franc No 010 grapes showed particularly interesting attributes for the production of high quality wines.

## Introduction

Grapevine (*Vitis vinifera* L.) is considered as the most important fruit crop in the world. Along with that, viticulture and enology play an important role in the economy of many countries [1]. The grapes represent a significant component of the human diet and wine industry because of their richness in phenolics. These compounds are secondary metabolites responsible for distinct functions in plants regarding the protection against biotic and abiotic environmental stress [2]. Different classes of soluble polyphenols are differently distributed in a berry; hydroxycinnamates (HCA), phenolic acids (PA) and proanthocyanidins are contained in berry pulp, skins are rich in anthocyanins, flavonols, HCA, PA and flavan-3-ols, while seeds contain flavan-3-ol monomers and tannins (polymeric flavan-3-ols) [3]. Among them anthocyanins isolated from the red grape berries (*Vitis vinifera* L.) have the simplest chemical structure of all the anthocyanins found in higher plants [4]. Their content in grapes is associated with some genetic factors (cultivar, clone), environmental and viticulture practices (light exposure, temperature, irrigation and nitrogen availability) [5]. Control of the ripening timing, berry size, and coloration, acidity and the relative selection of volatile and non-volatile aroma (polyphenols) and flavor compounds in wine grape cultivars are major concerns of viticulturists. All the above justify the scientific attention given to the development and maturation of grape berries [6]. The studies on wine polyphenols are particulary interesting due to their importance in evaluating the potential of different grape varieties and obtaining wines with distinctive or improved characteristics [7]. In addition, the interest in wine phenolics is still increasing because of their antioxidant and free radical-scavenging properties, supported by the positive effects on cardiovascular diseases, cancer, diabetes, and others [8].

In the Republic of Serbia, the viticulture and enology have a long tradition which dates back to Roman times. Merlot and Cabernet Franc are among the most important red grape varieties grown in Serbia and used for the production of high quality wines. These cultivars, originated in France, are successfully introduced in many regions with different climatic conditions, and are traditionally used for the production of famous Bordeaux wine [9]. Merlot is characterized with phenolic compounds of strong antioxidant potential inherited from Cabernet Franc (father) and early maturation and fertility from Magdeleine Noire des Charentes (mother) [10]. The plantations of these two cultivars in Serbia have a heterogeneous population [9, 11]. Therefore, the clonal selection is very important for obtaining the clones with better characteristics than the population. The complex selection criteria encompass traits such as grape yield, sugar content in grapes, the content of polyphenolic compounds in the grape and the quality of the produced wines [12]. The performances of a clone vary with environmental conditions due to clone-environment interactions [13]. For varieties with great genetic diversity, clonal selection is a major issue in the production of quality wines, as different clones of a same variety may vary in their productive properties and ability to give wines of different organoleptic properties [14]. The understanding of grape and wine phenolics is an increasingly important requirement for managing wine styles efficiently.

To obtain substantial knowledge on the quality of Merlot (No 022, 025 and 029) and Cabernet Franc (No 02, 010 and 012) clones, recently developed in Serbia, this work aimed to evaluate the phenolic compounds and radical scavenging activity of both grapes and corresponding wines of aforementioned clones. The mother grapes and wines were used as the relevant standards. Special attention was given to polyphenolic composition of grapes and respective wines, due to antioxidant properties of polyphenolics supported by their positive effects to human health. This is the primary report on the phenolic profile and radical scavenging activity of grape of Merlot and Cabernet Franc clones, selected for the first time in Serbia.

## Materials and Methods

### Plant material and growth conditions

Merlot clones, namely No 022, 025 and 029, and Cabernet Franc No 02, 010 and 012 (the last phase of clonal selection), were grown at the experimental field "Radmilovac" of the Faculty of Agriculture (Grocka viticultural region) with geographical coordinates 44° 45' N/20° 35' E, at an altitude of 153 m. The locality is situated in the area of Šumadija and Velika Morava, a sub-region of Belgrade, with modified moderate continental climate conditions. The terrain exposition is southwest, while the rows are directed southeast-southwest. The aforementioned clones and standards (mother vines) were grown under the same conditions. Standard ampelotechnical measures were applied, with no irrigation. During the year of the study (2013), meteorological conditions in the Grocka viticultural region were favorable. The average temperature in growing season (April-October) was 19.4°C, while the amount of precipitation was 320.9 mm.

### Chemicals

Methanol (HPLC grade) and acetonitrile (HPLC grade), as well as formic acid, ethyl acetate, and Folin-Ciocalteu reagent were purchased from Merck (Darmstadt, Germany). 2,2-Diphenyl-1-picrylhydrazyl˙ (DPPH˙) was purchased from Fluka AG (Buch, Switzerland) while syringe filters (13 mm, PTFE membrane 0.45 μm) were purchased from Supelco (Bellefonte, PA, USA). Trolox and standards of polyphenolics (gallic, protocatechuic, *p*-hydroxybenzoic, chlorogenic, caffeic, ferulic rosmarinic and *p*-coumaric acids; aesculin, epigallocatechin, catechin, epicatechin, gallocatechin gallate, catechin gallate, epigallocatechin gallate, rutin, morin, naringin, apigenin, and luteolin) used for UHPLC-MS/MS analysis were purchased from Sigma‐Aldrich (Steinheim, Germany). Ultrapure water (TKA Germany MicroPure water purification system, 0.055 μS cm^-1^) was used to prepare all standard solutions and dilutions. All other reagents were of analytical grade.

### Preparation of grape sample extracts

The grapes of Merlot and Cabernet Franc (standards and corresponding clones) were collected at three phases of grape development: green berries (the 14^th^ of June, 2013), véraison (the 14^th^ of August, 2013) and full maturity (the 14^th^ of October, 2013). Glyco-acidimetric index (GAI) was calculated as the ratio between sugar content and total acids. Grape samples were prepared according to modified literature method [15]. Frozen grape berry samples were homogenized and five grams of each sample was mixed with 50 mL of methanol containing 0.1% HCl. The extraction was carried out during 1h on a magnetic agitator, at room temperature. The extracts were placed in the dark at 4°C for 24 h, filtered and the clear supernatants were collected. The supernatants collected from three extractions were evaporated to dryness (by rotary evaporation under reduced pressure at 40°C) and the mixture methanol/water (60/40) was added to *ca*. 50 mL. Before further analysis all the extracts were filtered through 0.45 μm membrane filters (Syringe Filter, PTFE, Supelco).

### Microvinification

At the stage of full maturity samples of the studied grapes were harvested and immediately processed in laboratory conditions, using microvinification technique. For the purpose of microvinification, 20 kg of grapes, both standard and clones, were used. Crushing was done manually using a grape crusher with rollers and supplement for stem removal (for separating the grapes from the stems). In the must 100 mg L^-1^ potassium metabisulfite was added. The spontaneous fermentation took place in 20 L glass bottles at 20–25°C. Upon completion of the fermentation, the wine was decanted from the lees and bottled. The bottled wine was stored under 10°C for two months until chemical analysis [16].

### Analysis of classical oenological parameters

After the aging in a bottle classic wine parameters of standards and clones were analysed using the methods of the International Organisation of Vine and Wine: the alcoholic strength (ethyl alcohol) was measured with a pycnometer in a distillate obtained by the distillation of a certain volume of wine; total extract was determined by a specific densiometric method using a 50 mL pycnometer; total acids were measured by titration with NaOH up to pH 7 using bromothymol blue as indicator; pH was determined by potentiometry; volatile acids were separated from the wine by steam distillation and titrated with NaOH using phenolphthalein as an indicator; and the relative density was determined by picnometry (densitometry) [17].

### Determination of total phenolic content (TPC)

The total fenolic content in samples was determined using a modified version of the Folin–Ciocalteu method described in literature, using gallic acid as standard [15]. Briefly, 0.5 mL of the extracts and 0.5 mL ultrapure water were mixed with 2.5 ml of Folin-Ciocalteu reagent (100 ml L^-1^). The mixture was incubated for 5 min at room temperature. Subsequently, 2 mL of sodium carbonate (75 g kg^-1^) was added. After incubation during 2 h at room temperature, the measurements of the absorbance (at 765 nm) were performed on a GBC UV-Visible Cintra 6 spectrophotometer. The concentrations of gallic acid were in the range of 20−100 mg L^-1^. A mixture of water and reagent was used as a blank. TPC values were expressed as gram gallic acid equivalent (GAE) per kg of frozen weight (FW) of grape and per L of wine. All measurements were done in triplicate and the results were expressed as mean values ± standard deviation (*SD*).

### Determination of the radical-scavenging activity (RSA)

Radical scavenging activity was determined using DPPH radical solution by a slightly modified literature methods [15,18]. The extracts (0.1 mL) were mixed with 4 mL of methanol solution of DPPH^•^ (71 μmol L^-1^), and the mixtures were left to stand for 1 h in the dark, at room temperature. The absorbance was measured at 515 nm. RSA was calculated as a percentage of DPPH· discoloration using the equation:

$$RSA\;(\%)=\frac{\left(A_{DPPH}-A_{sample}\right)}{A_{DPPH}}\times 100$$

where *A*_*DPPH*_ is the absorbance of methanol solution of DPPH·, *A*_*sample*_ is the absorbance in the presence of samples. Trolox was used as standard (concentrations 100, 200, 300, 400, 500, and 600 μmol L^-1^). The calibration curve was displayed as a function of the percentage of inhibition of DPPH radical. The results were expressed as milimoles of Trolox equivalents per kg of frozen weight of grape (mmol TE kg^-1^ FW) and per L of wine (mmol TE L^-1^). The results were presented as mean values of three measurements ± standard deviation (*SD*).

### Determination of total anthocyanin content (TAC)

The total anthocyanin content was determined by the pH-differential method as described by Pavlović et al. [15]. Grape extracts and wines were diluted with buffers of pH 1.0 (KCl, 0.025 mol L^-1^) and pH 4.5 (NaOAc/HOAc, 0.4 mol L^-1^). The absorbances were measured at 510 and 700 nm against blank cell filled with distilled water. TAC was calculated using formula:

$$TAC=\frac{{\text{A}}_{\text{tot}}\times \text{MW}\times \text{DF}\times 1000}{\left(\varepsilon \times \text{l}\right)}$$

$$A_{tot}={\left(A_{510}-A_{700}\right)}_{pH_{1.0}}-{\left(A_{510}-A_{700}\right)}_{pH4.5}$$

where A_tot_ is the absorbance, MW is the molecular weight (MW = 493.2 g mol^-1^ for malvidin-3-*O*-glucoside), DF is the dilution factor, l is the cuvette pathlength (l = 1cm), ε is molar absorptivity (ε = 28000 L mol^-1^cm^-1^ for malvidin-3-*O*-glucoside). The results were expressed as mg malvidin-3-*O*-glucoside (mal-3-glu) equivalents per kg of frozen weight of grape and per 1 L of wine, respectively. The measurements were expressed as mean values ± standard deviation (*SD*).

### UHPLC–DAD MS/MS analysis of polyphenolic compounds

Separation and quantification of compounds of interest in each sample were performed using a Dionex Ultimate 3000 UHPLC system (Thermo Fisher Scientific, Bremen, Germany) equipped with a diode array detector (DAD) and connected to a triple-quadrupole mass spectrometer. The elution was performed at 40°C on a Syncronis C18 column (100 x 2.1 mm, 1.7 lm particle size). The mobile phase consisted of (A) 0.01% aqueous acetic acid solution and (B) acetonitrile, with flow rate 0.4 mL min^-1^, in the following gradient: 0.0–2.0 min 5% B, 2.0–12.0 min from 5% to 95% (B), 12.0–12.1 min from 95% to 5% (B), then 5% (B) for 3 min. The detection wavelength was set to 254 nm and the injection volume was 5 μL. A 1 mg mL^-1^ stock methanolic solution of a mixture of polyphenolics was prepared. The stock solution was diluted with ultrapure water yielding working solution of concentrations 0.010, 0.050, 0.100, 0.250, 0.500, 0.750, and 1.0 mg mL^-1^. Calibration curves revealed good linearity, with *R*^2^ values exceeding 0.99 (peak areas vs. concentration). Quantitative analysis was acquired in negative ionization mode on a TSQ Quantum Access Max triple-quadrupole mass spectrometer (Thermo Fisher Scientific, Basel, Switzerland), equipped with heated electrospray ionization (HESI) source. Vaporizer temperature was set at 200°C and the ion source settings as follows: spray voltage 5 kV, sheet gas (N_2_) pressure 40 AU, ion sweep gas (N_2_) pressure 1 AU and auxiliary gas (N_2_) pressure 8 AU, capillary temperature 300°C, and skimmer offset 0 V. The mass spectrometry data were acquired in negative ion mode, in the *m/z* range from 100 to 1000. Multiple mass spectrometric scanning modes, including full scanning (FS), and product ion scanning (PIS), were conducted for the qualitative analysis of the targeted compounds. The collision-induced fragmentation experiments were performed using argon as the collision gas, and the collision energy varied depending on the compound. The time-selected reaction monitoring (tSRM) experiments for quantitative analysis were performed using two MS^2^ fragments for each phenolic compound that were previously defined as dominant in the PIS experiments [19]. Xcalibur software 2.2 (Thermo Fisher, Bremen, Germany) was used for instrument control. The phenolics were identified by direct comparison with commercial standards. The total amount of each compound was evaluated by the calculation of the peak areas and expressed as mg kg^-1^.

### Statistical Analysis

Tukey’s test was used to detect the differences (p ≤ 0.05) among the mean values. Statistical analyses were performed by NCSS software package (www.ncss.com) and the statistical program MS Excel (Microsoft Office 2007 Professional). Principal Component Analysis (PCA) was realized using the PLS_Tool Box software package for MATLAB (Version 7.12.0). All data were group-scaled prior to PCA. The singular value decomposition algorithm (SVD) and a 0.95 confidence level for *Q* and Hotelling *T*2 limits for outliers were chosen.

## Results and Discussion

### TPC, TAC and RSA in grapes and wines

Total phenolic content, total anthocyanin content, and radical scavenging activity of eight grape samples from two cultivars (Merlot and Cabernet Franc) were measured in three different periods of grape development. The growth of seeded grapes is typified by three clearly stages: the first stage (rapid berry growth) is a period immediately after bloom and is characterized by organic acid accumulation. The second stage is slow or no growth of berries when they remain firm, but begin to lose chlorophyll. The start of the third stage, called véraison (berry softening) corresponds to the onset of ripening. This period is characterized by different biochemical and physiological changes like softening and coloring. The development of grape berries is associated with changes in size and composition [20]. The onset of ripening (véraison), signals the beginning of significant changes in metabolism which, among other things many, include accumulation of sugar and synthesis of anthocyanins. In spite of the fact that these changes occur within each berry, the significant variation between berries at harvest are present. The reason for this lies in fact that individual berries, even those on the same bunch, do not ripen synchronously [21]. Based on the results of our study for Merlot and Cabernet Franc grape it was noticed that standards and corresponding clones expressed statistically significant differences in respect to the content of polyphenols, anthocyanins, and antioxidant capacity radical scavenging activity in all three phases of berry development (Table 1). TPC for Merlot grapes varied from 3.71 to 29.79 g GAE kg^-1^ FW, whereas for Cabernet Franc it was in range from 2.76 to 42.82 g GAE kg^-1^ FW, depending on the berry development stage and clone. In the beginning of berry growth, significantly higher TPC values were found in grapes of Merlot standard and clone No 022 compared to clones No 025 and No 029.

**Table 1:** Total phenolic contents, total anthocyanin contents, and radical scavenging activity of Merlot and Cabernet Franc grapes.

	14^th^ of June			14^th^ of August			14^th^ of October		
	TPC (g GAE kg^-1^)	TAC (mg mal 3-glu kg^-1^)	RSA (mmol TE kg^-1^)	TPC (g GAE kg^-1^)	TAC (mg mal 3-glu kg^-1^)	RSA (mmol TE kg^-1^)	TPC (g GAE kg^-1^)	TAC (mg mal 3-glu kg^-1^)	RSA (mmol TE kg^-1^)
**Merlot** standard	28.30±0.14^*a*^	/	181.03±2.56^*a*^	12.33±0.21^*a*^	304.33±0.89^*a*^	80.91±2.85^*a*^	3.71±0.03^*c*^	698.38±0.15^*b*^	28.87±0.25^*d*^
No 022	29.79±0.46^*a*^	/	173.29±3.66^*b*^	11.73±10^*a*^^*b*^	176.64±1.98^*c*^	72.24±1.48^*c*^	4.38±0.01^*b*^	721.19±1.02^*a*^	46.21±2.96^*b*^
No 025	25.72±0.11^*b*^	/	129.27±1.14^*d*^	11.18±0.37^*b*^	135.36±1.10^*d*^	75.46±0.00^*b*^	7.32±0.11^*a*^	386.82±1.14^*c*^	79.74±1.32^*a*^
No 029	23.70±0.50^*b*^	/	136.33±2.82^*c*^	8.97±0.05^*c*^	233.73±1.43^*b*^	78.67±2.47^*a*^	3.81±0.03^*c*^	359.00±0.68^*d*^	41.37±1.49^*c*^
**Cabernet Franc** standard	13.41±0.04^*d*^	/	119.06±2.54^*d*^	9.32±0.07^*b*^	238.70±2.67^*a*^	57.53±7.82^*b*^	2.76±0.04^*c*^	416.72±0.96^*d*^	26.81±3.38^*d*^
No 02	16.83±0.06^*c*^	/	141.61±0.25^*c*^	10.63±0.01^*b*^	52.98±0.50^*d*^	77.84±3.20^*a*^	7.40±0.06^*b*^	488.07±2.53^*c*^	56.81±1.16^*b*^
No 010	25.17±0.17^*b*^	/	163.11±2.43^*b*^	11.81±0.01^*a*^	63.03±0.75^*c*^	77.21±2.82^*a*^	10.89±0.35^*a*^	1668.18±1.87^*a*^	80.48±0.26^*a*^
No 012	42.82±0.07^*a*^	/	191.73±3.02^*a*^	12.16±0.14^*a*^	138.27±3.01^*b*^	77.65±0.78^*a*^	6.97±0.04^*b*^	1075.07±0.99^*b*^	50.16±2.08^*c*^

TPC—Total phenolic content; TAC- total anthocyanin contents; RSA—radical scavenging activity

^a^Values represent means of triplicate determinations ± standard deviation.

Different letters in same row for Merlot/Cabernet Franc denote a significant difference according to Tukey’s test, *p*<0.05.

All clones showed distinct radical scavenging activities compared to corresponding standards. Merlot standard had the highest RSA value (181.03 mmol TE kg^-1^) while clone No 025 had the lowest RSA (129.27 mmol TE kg^-1^) compared to clones No 025 and 029. In Cabernet Franc grapes the highest/lowest contents of polyphenols were found in clone No 012 and standard, respectively. The following trends were observed in véraison: the lowest TPC and RSA were found in Merlot No 029 (8.97 g GAE kg^-1^ FW) and No 022 (72.24 mmol TE kg^-1^ FW), while the standard had the highest values of both parameters. Cabernet Franc clones had significantly higher TPC and RSA compared to the standard, except No 02. At full maturity of grapes, within the variety, Merlot No 025 and Cabernet Franc No 010 stood out with the highest TPC (7.32 g and 10.89 GAE kg^-1^) and RSA (79.74 and 80.48 mmol TE kg^-1^) respectively (Table 1). According to literature data secondary metabolites are variable among clones [12]. Consequently, it can be concluded that the grapes of these two clones are a better sources of natural antioxidants than corresponding standards and other studied clones. The distinct difference observed in TPC values of standard and clones of both varieties suggests that even a slight genetic alteration can significantly affect grape phenolic content. The results obtained for Cabernet Franc clones are consistent with the findings of Hogan et al. who found the significant differences in TPC and DPPH. scavenging activities between two Cabernet Franc clones (namely 1 and 313) [22]. TPC values obtained for both Merlot clones and standard, as for all Cabernet Franc clones are higher compared to literature data [23, 24]. Both grape varieties recorded a decrease (2–6 times) in TPC from the phase of fruit set to ripening (Table 1), which is in accordance with previous results [25]. This pattern was similar for both Merlot and Cabernet Franc corresponding clones. During the grape ripening berries change in their size and approximately double in size between véraison and harvest. The compounds accumulated in the grape berry during the first stage of development remain at harvest, but their concentration is significantly reduced due to the increase in berry volume. Also, the major soluble phenolic classes found in the grape berry share phenylalanine as the common biosynthetic precursor, and most of them exhibit patterns of accumulation and subsequent decline during ripening, suggesting their degradation or utilization in biosynthesis of other compounds [26]. Taken all together, the decline of TPC and RSA for both Merlot and Cabernet Franc grape samples during the grape development in our study was expected. The high correlation was found between TPC and RSA for green berries and mature grapes, suggesting that RSA was mainly dependant on the phenolic compounds in these stages of berry development.

In red grapes, anthocyanins, which are responsible for the red color of grape and wine, are synthesized in the skin and their accumulation initiates at véraison (the onset of ripening) [21,27]. The concentration of anthocyanins is inherited as a quantitative trait controlled by many genes and influenced by developmental stages of berries [5]. As expected, the anthocyanins were not found at the beginning of berries development, while their concentrations significantly increase in all studied samples of mature grapes. The accumulation rate differed among the clones. The greatest amount of anthocyanins in véraison was found in both Merlot and Cabernet Franc standards, while the lowest were in Merlot No 025 and Cabernet Franc No 02. It is known that this initial phase of anthocyanins accumulation is strongly correlated with sugar accumulation and mainly influenced by vine vegetative and photosynthetic conditions, whereas the second one is detached from sugars and strongly affected by climatic conditions [5]. Mature grape of Merlot No 022 had the highest total anthocyanin content (721.19 mg mal 3-glu kg^-1^ FW) whereas Merlot No 029 had the lowest (359.00 mg mal 3-glu kg^-1^ FW) showing a 2-fold difference (Table 1). The results obtained in this study for Merlot standard and No 022 are consistent with literature [28]. In all Cabernet Franc grapes analysed, TAC varied from 416.72 to 1668.18 mg mal 3-glu kg^-1^ FW, with significant differences between standard and clones. The highest TAC was measured in clone No 010, 4-fold difference compared to standard (Table 1). The studied Cabernet Franc grape samples had higher concentrations of anthocyanins than reported by other authors [24,28].

In our previous study, the results obtained for TPC and TAC in Merlot wines suggested that clones significantly differed among themselves [16]. The same was observed for Cabernet Franc wines, as shown in Table A in S1 File. In studied Cabernet Franc wine samples 1.11 g GAEL^-1^ to 1.58 g GAEL^-1^ of total phenols and 62.71–210.31 mg mal 3-glu L^-1^ of total anthocyanins were measured. The results that we obtained for total phenol content in Merlot and Cabernet Franc wines were in accordance with literature data; it usually varies from 160 to 3200 mg L^-1^ expressed as gallic acid [29]. The anthocyanins are unstable and easily subject to degradation. One of the factors that affects their stability is the pH value. The concentration of the flavylium form, the major form of anthocyanins in wines, declines rapidly as the pH rises [30]. The lower pH of Cabernet Franc wines might be the reason for the higher anthocyanin content in all Cabernet Franc wines compared to Merlot wines. It can be observed that the levels of anthocyanins in Merlot and Cabernet Franc wines and respective grapes were not particularly correlated [30]. Merlot No 025 and Cabernet Franc No 010 stood out with the highest values of RSA (11.01 and 11.02 mmol TE L^-1^, respectively).

### Classical enological parameters

The results of the chemical composition of studied wines are summarized in Table B in S1 File. In general, all wines showed high amount of total acidity (ranged from 6.30 to 7.24 g L^-1^), which is important for the microbial stabilization and the freshness of these wines. Real acidity (pH) was lower in Cabernet Franc (3.09–3.32) than in Merlot (3.46–3.47) wines. Cabernet Franc wines (13.04–13.22 vol %) had slightly higher alcohol content compared to Merlot wines (12.41–12.46 vol %). This might be the consequence of the difference in grape maturity, observed through the glyco-acidimetric index (GAI, data not shown). GAI was higher in Cabernet Franc (3.00–3.44) than in Merlot grapes (2.85–2.96). The results obtained for the total acidity and sugar content were within the limits for the studied cultivars [31].

### Analysis of the polyphenolic compounds in grapes and wines

The evaluation and identification of polyphenol composition of grape is an important factor in estimating its oenological potential [32]. The balanced levels of phenols and adequate content of sugars and acids in grapes are a prerequisite for making high quality wines. In this study the total of 19 compounds were quantified in all studied grape samples using the available standards (Tables 2 and 3). The phenolic acids (gallic, *p*-hydroxybenzoic, chlorogenic, caffeic, ferulic and rosmarinic), catechin gallate, rutin and morin were quantified in all analyzed Merlot grapes. As for Cabernet Franc grapes, gallic, *p*-hydroxybenzoic, caffeic, *p*-coumaric acids, aesculin, catechin gallate, rutin, and morin were found in all studied samples. The concentrations of individual polyphenols varied depending on studied samples and berry development stages. Generally, the content of most phenolics decreased from the phase of fruit set to full ripeness. It was worth mentioning that the highest concentrations of catechin and epicatechin (the most abundant in grapes of all clones) were in véraison, while at full ripeness they were substantially lower, which is consistent with literature [33]. These phases could refer to a period of accumulation and a period of decline of these flavan-3-ols.

**Table 2:** Polyphenolic content in Merlot grapes at three stages of berry development.

mg kg^-1^ FW	14^th^ of June				14^th^ of August				14^th^ of October			
	Standard	No 022	No 025	No 029	Standard	No 022	No 025	No 029	Standard	No 022	No 025	No 029
***Hydroxybenzoic acids***												
Gallic acid	30.29	3.90	2.74	2.18	2.94	2.46	3.71	2.88	5.94	3.48	2.68	1.78
Protocatechuic acid	0.09	0.25	nd	0.02	0.06	0.09	0.01	0.20	nd	nd	nd	0.01
*p*-Hydroxybenzoic acid	11.93	12.41	13.69	7.45	4.65	1.84	4.55	4.05	2.11	0.06	2.67	1.14
***Hydroxycinnamic acids***												
Chlorogenic acid	0.76	0.60	0.48	0.64	0.39	0.43	1.67	0.41	0.55	0.49	2.43	0.39
Caffeic acid	3.64	1.04	0.19	0.47	0.13	0.11	0.26	0.49	0.17	0.09	0.29	0.11
Ferulic acid	4.65	4.48	1.27	1.41	nd	1.04	1.20	1.60	nd	1.01	1.20	1.11
Rosmarinic acid	0.27	0.17	0.14	0.14	0.12	0.11	0.57	0.14	0.16	0.17	0.91	0.12
*p*-Coumaric acid	0.16	nd	0.25	nd	0.14	0.03	0.07	0.29	0.32	0.13	0.10	0.07
***Coumarins***												
Aesculin	2.00	2.14	0.25	1.09	0.41	0.44	0.38	0.39	0.46	0.27	nd	0.40
***Flavan-3-ols***												
Epigallocatechin	1.11	1.39	nd	1.29	1.26	1.21	1.18	nd	nd	nd	1.26	nd
Catechin	52.15	78.71	17.46	82.59	119.88	123.64	66.41	11.17	nd	24.19	52.20	11.14
Epicatechin	1.56	1.32	0.67	1.35	28.15	27.00	20.42	3.96	nd	9.19	13.50	7.31
Gallocatechin gallate	5.01	10.80	nd	7.99	nd	4.10	3.81	6.56	nd	4.16	3.69	nd
Catechin gallate	5.60	11.35	4.97	15.52	25.05	29.35	20.01	5.84	0.34	4.62	6.84	2.99
Epigallocatechin gallate	1.55	2.58	nd	0.36	nd	nd	0.71	nd	nd	0.40	0.61	nd
***Flavonols***												
Rutin	24.12	28.60	0.60	18.70	7.61	11.71	9.94	5.07	1.99	5.05	2.49	2.81
Morin	10.34	3.51	0.45	0.69	3.52	6.65	4.99	13.33	0.65	0.53	0.46	1.15
***Flavanons***												
Naringin	0.30	0.23	nd	nd	nd	0.28	0.22	0.22	nd	nd	nd	nd
***Flavons***												
Apigenin	0.34	0.25	0.22	0.22	0.23	0.22	0.24	0.24	nd	nd	nd	nd

**Table 3:** Polyphenolic content in Cabernet Franc grapes at three stages of berry development.

mg kg^-1^ FW	14^th^ of June				14^th^ of August				14^th^ of October			
	Standard	No 02	No 010	No 012	Standard	No 02	No 010	No 012	Standard	No 02	No 010	No 012
***Hydroxybenzoic acids***												
Gallic acid	2.84	7.96	5.79	7.65	4.17	4.40	3.47	4.03	0.85	5.36	4.56	2.75
Protocatechuic acid	0.02	0.22	nd	nd	nd	nd	0.02	0.08	0.07	0.01	nd	0.10
*p*-Hydroxybenzoic acid	17.84	10.58	14.06	6.28	4.36	7.18	6.85	4.42	2.32	3.46	2.13	0.91
***Hydroxycinnamic acids***												
Chlorogenic acid	1.10	0.66	0.61	0.73	0.37	0.39	0.39	nd	0.98	0.35	0.51	0.39
Caffeic acid	0.27	0.56	0.78	1.48	0.13	0.12	0.08	0.08	0.23	0.16	0.14	0.10
Ferulic acid	1.61	2.18	6.12	4.64	1.08	1.08	1.17	1.35	1.06	nd	1.10	1.10
Rosmarinic acid	0.34	0.23	0.20	0.26	0.12	0.12	nd	nd	0.38	0.12	0.15	nd
*p*-Coumaric acid	0.24	0.89	0.26	0.13	0.32	0.06	0.12	0.44	0.23	0.12	0.11	0.10
***Coumarins***												
Aesculin	0.26	0.26	0.82	1.78	0.38	0.27	0.32	0.37	0.31	0.39	0.42	0.33
***Flavan-3-ols***												
Epigallocatechin	nd	1.37	1.69	1.34	1.04	1.38	1.51	1.42	nd	nd	1.33	1.04
Catechin	10.68	24.72	80.10	81.95	20.23	111.19	88.83	115.85	nd	10.79	39.83	17.63
Epicatechin	3.81	1.66	2.37	1.70	4.83	30.03	25.30	31.15	nd	4.36	10.77	7.16
Gallocatechin gallate	3.73	4.63	17.44	10.31	5.09	4.84	4.06	4.42	nd	nd	3.96	nd
Catechin gallate	2.11	10.90	28.78	10.57	11.52	38.05	29.14	26.67	0.50	7.98	9.06	3.65
Epigallocatechin gallate	0.41	0.49	3.15	1.48	0.31	nd	nd	nd	0.32	nd	nd	nd
***Flavonols***												
Rutin	0.45	1.65	18.93	23.09	12.92	9.82	6.71	4.11	1.29	1.33	5.05	2.04
Morin	0.34	1.06	1.35	4.78	4.82	1.96	1.34	1.48	0.48	0.77	1.61	0.78
***Flavanons***												
Naringin	0.24	0.21	0.23	0.23	0.21	nd	nd	nd	nd	nd	nd	nd
***Flavons***												
Apigenin	nd	0.24	0.23	0.30	0.22	0.22	nd	0.24	nd	0.22	0.22	nd

The grape composition is a very complex trait and it varies among genotypes under given growing conditions [5]. Regarding the chemical composition of both Merlot and Cabernet Franc mature grapes our study revealed that the most abundant phenolics were gallic acid, *p*-hydroxybenzoic acid, ferulic acid, epigallocatehin, catechin, epicatechin, gallocatechin gallate, catechin gallate, and rutin. The lower content of gallic acid in the grapes of Merlot clones compared to standard might be explained by the higher content of its esters gallocatechin gallate, catechin gallate and epigallocatechin gallate in respective grapes. In Merlot standard sample only catechin gallate was found of all quantified flavan-3-ols. Catechin, epicatechin and catechin gallate were found in grape of all three Merlot clones. The leading phenolic compounds were catechin and epicatechin and No 025 stood out with the highest concentration (52.20 mg kg^-1^ FW and 13.50 mg kg^-1^ FW, respectively). Besides, clone No 025 stood out with the highest content of *p*-hydroxybenzoic, chlorogenic, caffeic, ferulic and rosmarinic acids; epigallocatechin, catechin gallate and epigallocatechin gallate. This clone differed from both the standard and other two clones in respect of the presence of epigallocatechin (1.26 mg kg^-1^ FW) in mature grapes (Table 2). Briefly, catechin and epicatechin were present in grape of studied Merlot clones but not in Merlot standard. Catechin gallate was found in mature grape of all four Merlot samples, while gallocatechin gallate and epigallocatechin gallate only in Merlot No 022 and No 025 grapes. As opposed to our research results, Mattivi et al. did not determine catechin gallate, gallocatechin gallate and epigallocatechin gallate in respective grape extracts of official Merlot clone (INRA 184) grown in three Italian vineyards [34].

The following findings were characteristic for polyphenol profile of mature grape of Cabernet Franc clones: compared to mother grape they had higher content of studied phenolics, except of chlorogenic, caffeic, rosmarinic and *p*-coumaric acids. Catechin and epicatechin were present in grape of all Cabernet Franc clones, although were not found in the standard. It was notable that gallocatechin gallate, epigallocatechin and epigallocatechin gallate were found only in clone No 010 (3.96 mg kg^-1^ FW), clones No 010 and 012 (1.33 and 1.04 mg kg^-1^ FW, respectively) and standard (0.32 mg kg^-1^ FW). Clone No 010 differed from standard and other two clones regarding the highest content of aesculin, epigallocatechin, catechin, epicatechin, gallocatechin gallate, catechin gallate, rutin and morin (Table 3). Besides, the enhanced content of some phenolics in grapes of Merlot No 025 and Cabernet Franc No 010 had impact on their antioxidant activity, which was found to be the highest for these clones. The content and the structure of flavanols in studied grape samples have led to differences in the composition which seem to play an important role in the nutritional and sensorial properties of the wines. The grape variety and other factors that affect the berry development (e.g. the edaphoclimatic conditions or geographical location) have a significant influence on the polyphenolic composition of wines, while the phenolic content of wine varies according to the grape variety and vintage [7]. In this study, gallic acid was the most prevailing acid in all wine samples, which is consistent with literature [22]. Its concentration ranged from 20.033 (No 025) to 28.887 mg L^-1^ (standard) and 18.395 (No 010) to 27.030 mg L^-1^ (standard) in Merlot and Cabernet Franc wines, respectively (Table C in S1 File). Galic acid is formatted mainly through the hydrolysis of flavonoid gallate esters, which explains significant amounts of gallic acid in studied wines [35]. This acid greatly adds to the antioxidative capacity of red wines [36]. Far less abundant was protocatechuic acid, while caffeic and *p*-coumaric acids were the most represented among hydroxycinnammic acids, which is in agreement with literature [37]. It was observed that *p*-hydroxybenzoic was not found only in Cabernet Franc No 010. In Merlot wines the highest concentrations of catechin (4.307 mg L^-1^) and epicatechin (1.593 mg L^-1^) were found in clone No 029, while the highest concentration of gallocatechin gallate (1.938 mg L^-1^) was observed in clone No 025. In Cabernet Franc wines, the most abundant flavan-3-ols were catechin (8.091 mg L^-1^) and gallocatechin gallate (1.514 mg L^-1^) in clone No 012, and epicatechin (2.337 mg L^-1^) in No 010. The results of this study regarding the contents of catechin are within literature reported values [38]. Merlot No 025 and Cabernet Franc No 012 had the highest amounts of aesculin within the variety (0.096 and 0.066 mg L^-1^, respectively). Concentrations of rutin ranged from 0.027 (Merlot No 022) to 0.037 mg L^-1^ (Cabernet Franc 010 and 012) (Table C in S1 File). The polyphenolic profile of both Merlot and Cabernet Franc wine samples differed from corresponding grapes regarding the composition and content of individual compounds, most probably due to their concentration in the berries, different extractability during the maceration and the stage of ripeness. In addition, the modified composition and content of polyphenols in the studied wines was influenced by diverse reactions such as oxidation or copigmentation reactions that occur during the fermentation [7].

### Correlations between antioxidant activity and composition of phenolic compounds

Pearson correlation analysis was used to investigate relationships between TPC and RSA values (Table D in S1 File). The close correlation between TPC and RSA values obtained for green berries and full maturity (*r* = 0.830, *P* < 0.05 and *r* = 0.859, *P* < 0.005, respectively), suggests that the antioxidant activity of grapes in those phases of grape development is derived mainly from their phenolic compounds. Significant correlations between TPC and RSA values measured for wine samples were obtained (*r* = 0.897, *P* < 0.005). However, no evident correlation was found between the TPC and RSA for véraison phase. To further investigate the impact of the individual phenolic compounds identified in the extracts on the activity, the content of an individual phenolic compound was correlated with the results of the radical scavenging activity (Table D in S1 File). From these correlations, one can identify compounds that are potentially responsible for the antioxidant properties of the extracts. As for green berries, some phenolic compounds, such as ferulic acid, aesculin, and rutin showed high positive correlation with RSA (*r* = 0. 838, *r* = 0. 859 and *r* = 0. 838, respectively, *P* < 0.01). Similarly, several flavan-3-ols (catechin, epicatechin, epigallocatechin, and catechin gallate) typical of the samples in full maturity showed significant correlation with RSA values (*r* = 0. 923, *P* < 0.005; *r* = 0. 857, *P* < 0.01; *r* = 0. 814, *P* < 0.05; and *r* = 0. 911, *P* < 0.005, respectively). On the contrary, the contents of individual phenolics measured in véraison phase and wine samples did not show correlation with RSA values (Table D in S1 File).

### Principal component analysis (PCA)

**Fig 1:**
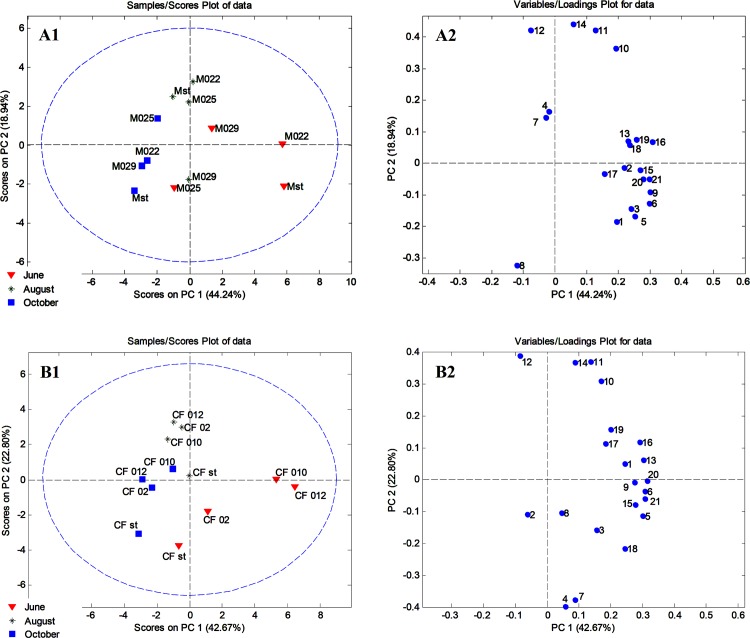
Principal component analysis (PCA) of Merlot and Cabernet Franc grapes. A1) PC scores plot of Merlot grape samples; A2) Loadings plot of Merlot grape samples; B1) PC scores plot of Cabernet Franc grape samples; B2) Loadings plot of Cabernet Franc grape samples.

PCA was carried out separately on Merlot (M) and Cabernet Franc (CF) grape samples Fig 1. Data that contained 12 objects (the number of grape samples) × 21 variables (quantified polyphenolics, TPC, and RSA) were processed using the covariance matrix with auto-scaling. PCA for Merlot grapes resulted in a five-component model that explained 91.09% of the total variance. The first principal component accounted for 44.24%, the second for 18.94% and the third component for 11.90% of the total variance.

The PCA correlation plot, Fig 1; A1, showed clustering of the Merlot grape samples into three distinctive groups according to phases of berry growth (June, August, and October). Grape of Merlot No 025 collected at the stage of full maturity differed from other Merlot samples collected at the same time, based on its higher contents of chlorogenic and rosmarinic acids Fig 1; A2. PCA obtained for Cabernet Franc grape samples resulted in six PCs explaining 93.39% of the total variance. The first principal component accounted for 42.67%, the second for 22.80% and the third component for 11.37% of the total variance. In Fig 1 (B1, PCA scatter plot) it is evident that Cabernet Franc grape samples were clustered in three main groups according to phases of grape development (June, August, and October). As for the Cabernet Franc samples collected at the stage of full maturity, Cabernet Franc standard (CF st.) differed from other grape samples according to higher content of *p*-coumaric acid Fig 1; B2.

## Conclusions

The first report on the phenolic profile of grape of Merlot and Cabernet Franc clones developed in Serbia, revealed significant differences between the grapes of standards and corresponding clones in respect to content of total polyphenols, anthocyanins, and radical scavenging activity. The mature grapes of studied clones were characterized with high TPC, TAC and RSA values and the notable correlations between TPC and RSA values for grape samples in two phases of grape development, green berries and full maturity, indicating that antioxidant activity of grapes was mainly dependent on the phenolic compounds. As for the wine samples, the significant correlations were also noticed between TPC and RSA values.

The great versatility was observed in the content of polyphenolic compounds among the studied clones. Merlot No 025 differed from both the standard and other two clones in respect to the highest concentrations of *p*-hydroxybenzoic, chlorogenic, caffeic, ferulic and rosmarinic acids, epigallocatechin, catechin, epicatechin, catechin gallate and epigallocatechin gallate. Cabernet Franc No 010 had the highest content of aesculin, epigallocatechin, catechin, epicatechin, gallocatechin gallate, catechin gallate, rutin and morin compared to standard and other two clones. Several flavan-3-ols (catechin, epicatechin, epigallocatechin, and catechin gallate) typical for the ripe grape of both Merlot and Cabernet Franc clones showed significant correlation with RSA values, indicating that these polyphenols might be the main contributors to the radical scavenging activity of the respective clones. The levels of individual phenolics were not consistently found in wines produced from a particular grape. Nevertheless, Merlot No 025 and Cabernet Franc No 010 were singled out in terms of the increased content of polyphenols in grapes. The findings pointed out to a good enological potential of these two clones, and technological utility of the results as the higher concentrations of phenolic compounds in grapes and in their resultant wines would produce higher quality wines.

The significance of the study is the characterization of new Merlot and Cabernet Franc clones (not yet commercial) with improved quality regarding the content of polyphenolics and antioxidant capacity compared to the mother vine. In the long term, it will lead to the introduction of the best ones in viticultural practice and production of high-quality wines.

## Supporting Information

S1 File The relevant data on Merlot and Cabernet Franc wines including: chemical composition, total phenolic content, total anthocyanin content, radical scavenging activity, polyphenolic contents, and grapes and wines: correlations between antioxidant activity and composition of phenolic compounds. (DOCX)
