# Supplementary material for: Impact of Clonal Variability on Phenolics and Radical Scavenging Activity of Grapes and Wines: A Study on the Recently Developed Merlot and Cabernet Franc Clones (Vitis vinifera L.)
Source: PLoS One. 2016 Oct 12;11(10):e0163823. doi: 10.1371/journal.pone.0163823 (PMC5061370; doi:10.1371/journal.pone.0163823)
Supplement: S1 File — (DOCX) [file pone.0163823.s001.docx]

**Supporting Information**

**Table A. Total phenolic content, total anthocyanin content, radical scavenging activity**

**of Merlot and Cabernet Franc wines**

|  | TPC  (g GAE L^-1^) | TAC  (mg mal 3-glu L^-1^) | RSA  (mmol TE L^-1^) |
| --- | --- | --- | --- |
| **Merlot**  standard | 1.28±0.01*^b*^* | 28.54±0.12*^c*^* | 9.68±0.40*^b^* |
| No 022 | 1.10±0.01*^d*^* | 24.31±0.87*^d*^* | 8.46±0.03*^c^* |
| No 025 | 1.46±0.00*^a*^* | 40.51±0.22*^a*^* | 11.01±0.19*^a^* |
| No 029 | 1.16±0.00*^c*^* | 35.58±0.00*^b*^* | 8.69±0.24*^c^* |
|  |  |  |  |
| **Cabernet Franc** standard | 1.12±0.00*^b^* | 86.66±0.14*^b^* | 7.58±0.03*^b^* |
| No 02 | 1.11±0.01*^b^* | 62.71±0.23*^d^* | 7.15±0.15*^b^* |
| No 010 | 1.41±0.02*^a^* | 210.31±1.01*^a^* | 11.02±1.01*^a^* |
| No 012 | 1.58±0.01*^a^* | 74.33±0.15*^c^* | 10.61±0.48*^a^* |

^*^Vujović et al., 2015*.*

**Table B. The chemical composition of Merlot and Cabernet Franc wines**

|  | Ethyl alcohol  (vol %) | Total extract  (g L^-1^) | Total acids  (g L^-1^) | Volatile acids  (g L^-1^) | pH | Relative density 20/20 |
| --- | --- | --- | --- | --- | --- | --- |
| **Merlot** standard | 12.41±0.08 | 26.80±0.08 | 7.23±0.06 | 0.67±0.04 | 3.47±0.04 | 0.9924±0.00 |
| No 022 | 12.42±0.06 | 27.06±0.04 | 7.23±0.06 | 0.66±0.03 | 3.46±0.03 | 0.9928±0.00 |
| No 025 | 12.46±0.07 | 26.80±0.07 | 7.24±0.06 | 0.68±0.03 | 3.47±0.03 | 0.9927±0.00 |
| No 029 | 12.44±0.08 | 26.84±0.08 | 7.23±0.06 | 0.67±0.04 | 3.46±0.03 | 0.9926±0.00 |
|  |  |  |  |  |  |  |
| **Cabernet Franc** standard | 13.10±0.12 | 27.33±0.27 | 6.45±0.26 | 0.76±0.10 | 3.32±0.06 | 0.9921±0.00 |
| No 02 | 13.04±0.16 | 27.34±0.17 | 6.36±0.18 | 0.67±0.08 | 3.12±0.06 | 0.9921±0.00 |
| No 010 | 13.22±0.11 | 26.74±0.16 | 6.30±0.13 | 0.76±0.06 | 3.28±0.09 | 0.9921±0.00 |
| No 012 | 13.08±0.20 | 26.76±0.20 | 6.44±0.09 | 0.64±0.07 | 3.09±0.05 | 0.9921±0.00 |

^a^Values represent means of triplicate determinations ± standard deviation.

**Table C.** **Polyphenolic content of Merlot and Cabernet Franc wines**

| mg L^-1^ | **Merlot wines** | | | | **Cabernet Franc wines** | | | |
| --- | --- | --- | --- | --- | --- | --- | --- | --- |
|  | Standard | No 022 | No 025 | No 029 | Standard | No 02 | No 010 | No 012 |
| ***Hydroxybenzoic acids*** |  |  |  |  |  |  |  |  |
| Gallic acid | 28.887 | 20.785 | 20.033 | 26.684 | 27.030 | 20.580 | 18.395 | 25.657 |
| Protocatechuic acid | 1.027 | 1.467 | 0.784 | 0.766 | 0.959 | 0.587 | 0.299 | 0.629 |
| *p*-Hydroxybenzoic acid | 0.215 | 0.403 | 0.552 | 0.111 | 0.106 | 0.132 | nd | 0.582 |
| ***Hydroxycinnamic acids*** |  |  |  |  |  |  |  |  |
| Chlorogenic acid | 0.138 | 0.191 | 0.102 | 0.110 | 0.041 | 0.117 | 0.096 | 0.194 |
| Caffeic acid | 2.436 | 1.533 | 1.288 | 1.344 | 0.445 | 1.111 | 0.852 | 1.238 |
| Ferulic acid | 0.374 | 0.192 | 0.268 | 0.336 | 0.158 | 0.154 | 0.271 | 0.284 |
| Rosmarinic acid | 0.034 | 0.027 | 0.030 | 0.035 | 0.012 | 0.023 | 0.027 | 0.031 |
| *p*-Coumaric acid | 1.122 | 0.947 | 0.874 | 1.156 | 2.121 | 0.796 | 0.746 | 0.961 |
| ***Coumarins*** |  |  |  |  |  |  |  |  |
| Aesculin | 0.068 | 0.066 | 0.096 | 0.074 | 0.030 | 0.056 | 0.059 | 0.066 |
| ***Flavan-3-ols*** |  |  |  |  |  |  |  |  |
| Epigallocatechin | 0.198 | 0.157 | 0.163 | 0.282 | 0.219 | 0.150 | 0.424 | 0.377 |
| Catechin | 4.244 | 2.002 | 1.558 | 4.307 | 4.129 | 2.533 | 7.379 | 8.091 |
| Epicatechin | 1.377 | 0.583 | 1.570 | 1.593 | 1.606 | 0.489 | 2.337 | 1.232 |
| Gallocatechin gallate | 1.663 | 1.710 | 1.938 | 1.820 | 0.503 | 0.649 | 1.197 | 1.514 |
| Catechin gallate | 0.051 | 0.037 | 0.032 | 0.031 | 0.039 | 0.029 | 0.038 | 0.036 |
| Epigallocatechin gallate | 0.077 | 0.057 | 0.058 | 0.081 | 0.173 | 0.050 | 0.060 | 0.044 |
| ***Flavonols*** |  |  |  |  |  |  |  |  |
| Rutin | 0.028 | 0.027 | 0.028 | 0.028 | 0.029 | 0.037 | 0.037 | 0.031 |
| Morin | 0.038 | 0.057 | 0.057 | 0.080 | 0.037 | 0.041 | 0.034 | 0.058 |
| ***Flavanons*** |  |  |  |  |  |  |  |  |
| Naringin | 0.040 | 0.037 | 0.042 | 0.039 | 0.086 | 0.039 | 0.032 | 0.037 |
| ***Flavons*** |  |  |  |  |  |  |  |  |
| Apigenin | 0.024 | 0.023 | 0.023 | 0.023 | 0.025 | 0.023 | 0.024 | 0.024 |
| Luteolin | nd | 0.040 | 0.040 | 0.040 | nd | 0.042 | 0.040 | nd |

**Table D. Correlation coefficients obtained for the relationships between RSA**

**and phenolic content (TPC and individual phenolic compounds)**

|  | **14^th^ of June** | **14^th^ of August** | **14^th^ of October** | **wine** |
| --- | --- | --- | --- | --- |
| **TPC** | **0.830***** | 0.481 | **0.895*** | **0.897*** |
| Gallic acid | 0.524 | -0.291 | 0.183 | -0.218 |
| Protocatechuic acid | 0.168 | 0.356 | -0.337 | -0.370 |
| *p*-Hydroxybenzoic acid | -0.492 | 0.257 | 0.260 | 0.409 |
| Chlorogenic acid | -0.205 | 0.006 | 0.425 | 0.223 |
| Caffeic acid | **0.711***** | 0.178 | 0.213 | 0.195 |
| Ferulic acid | **0.838**** | -0.142 | 0.313 | 0.600 |
| Rosmarinic acid | 0.061 | -0.022 | 0.390 | 0.543 |
| *p*-Coumaric acid | -0.284 | -0.221 | -0.630 | -0.461 |
| Aesculin | **0.859**** | -0.164 | -0.448 | 0.587 |
| Epigallocatechin | 0.658 | 0.012 | **0.814***** | 0.554 |
| Catechin | 0.684 | 0.476 | **0.923*** | 0.453 |
| Epicatechin | -0.270 | 0.545 | **0.857**** | 0.601 |
| Gallocatechin gallate | 0.539 | -0.308 | 0.683 | 0.596 |
| Catechin gallate | 0.252 | 0.368 | **0.911*** | 0.199 |
| Epigallocatechin gallate | 0.685 | -0.357 | 0.215 | -0.448 |
| Rutin | **0.838**** | -0.712 | 0.433 | -0.013 |
| Morin | **0.758***** | -0.031 | 0.406 | 0.042 |
| Naringin | 0.554 | -0.499 | / | -0.466 |
| Apigenin | **0.772***** | -0.080 | 0.524 | -0.085 |
| Luteolin | / | / | / | -0.024 |

******P* ≤ 0.005; ****** *P* ≤ 0.01; ******* *P* ≤ 0.05.
